# Identification and analysis of hub genes and networks related to hypoxia preconditioning in mice (No 035215)

**DOI:** 10.18632/oncotarget.23555

**Published:** 2017-12-21

**Authors:** Haiting Cheng, Can Cui, Shousi Lu, Binbin Xia, Xiaorong Li, Pinxiang Xu, Ming Xue

**Affiliations:** ^1^ Department of Pharmacology, Beijing Laboratory for Biomedical Detection Technology and Instrument, School of Basic Medical Sciences, Capital Medical University, Beijing 100069, China; ^2^ China Rehabilitation Research Center, Beijing 100068, China; ^3^ Department of Pharmacy, Beijing Stomatological Hospital, Capital Medical University, Beijing 100050, China

**Keywords:** hypoxia preconditioning, hub genes, microarray, network analyses

## Abstract

Hypoxia preconditioning is an effective strategy of intrinsic cell protection. An acute repetitive hypoxic mice model was developed. High-throughput microarray analysis was performed to explore the integrative alterations of gene expression in repetitive hypoxic mice. Data obtained was analyzed via multiple bioinformatics approaches to identify the hub genes, pathways and biological processes related to hypoxia preconditioning. The current study, for the first time, provides insights into the gene expression profiles in repetitive hypoxic mice. It was found that a total of 1175 genes expressed differentially between the hypoxic mice and normal mice. Overall, 113 significantly up-regulated and 138 significantly down-regulated functions were identified from the differentially expressed genes in repetitive hypoxic brains. Among them, at least fourteen of these genes were very associated with hypoxia preconditioning. The change trends of these genes were validated by reverse-transcription polymerase chain reaction and were found to be consistent with the microarray data. Combined the results of pathway and gene co-expression networks, we defined *Plcb1*, *Cacna2d1*, *Atp2b4*, *Grin2a*, Grin2b and Glra1 as the main hub genes tightly related with hypoxia preconditioning. The differential functions mainly included the mitogen-activated protein kinase pathway and ion or neurotransmitter transport. The multiple reactions in cell could be initiated by activating MAPK pathway to prevent hypoxia damage. Plcb1 was an important and hub gene and node in the hypoxia preconditioning signal networks. The findings in the hub genes and integrated gene networks provide very useful information for further exploring the molecular mechanisms of hypoxia preconditioning.

## INTRODUCTION

The maintenance of oxygen homeostasis in cells, tissues, organs and organisms is a complex process of integration and regulation, which is primarily dominated via changes in gene expression. Oxygen deprivation (hypoxia) is a great challenge in some conditions such as high plateau, deep sea, and aviation [1]. Hypoxia might be associated with many pathophysiological processes and diseases such as stroke, asthma, emphysema, angina pectoris, myocardial infarction and tumor [2]. Oxygen is indispensable for all aerobic life as the final electron acceptor in the cellular respiration chains [3]. Successful adaptation to hypoxia involves various changes in gene programs including cellular differentiation, metabolism, growth, aging and death. Since complicated genes and pathways are involved in the regulation of oxygen homeostasis, the molecular mechanisms that underpin these networks are not yet fully understood. Hypoxia preconditioning (HPC) is an effective strategy of intrinsic cell protection that developed during biological evolution [4]. HPC is triggered by the repetitive exposure of organisms, organs, tissues and cells to hypoxia, resulting in an increased acclimatization to subsequent exposure to severe hypoxia. The results indicated that the survival time of HPC mice was significantly longer than that of the normal mice, suggesting that HPC had a markedly protective effect against hypoxia damage [5–8]. Although the protective effect clearly exists and some investigations have been performed, the protective mechanisms of HPC still remain unclear, especially at molecular level of the genes involved.

High-throughput microarray analysis is an effective approach to profile gene expression and has been successfully applied to investigate crucial genes related to HPC [9, 10]. Because of the high sensitivity to hypoxia, the brain tissue has been used to identify the important genes related to HPC by microarray gene-chip and gene network analyses. The gene network analysis is a powerful tool and has been used to investigate the detailed interactions among genes [11, 12]. In the current study, the changes in gene expression levels were observed between the experimental group and control group by multiple sample analysis. The multiple bioinformatics analysis approaches have been used to construct the gene networks of HPC, and the hub genes in these networks have been identified. The expression analyses of entire genome combined with gene network analyses are expected to reveal the protective mechanisms for HPC. Here, the genes in the brain tissue of HPC group and control group were analyzed using the Affymetrix Gene 1.0 Array [11]. In addition, Real-time reverse-transcription polymerase chain reaction (RT-PCR) was used to validate the differential expression of genes identified by microarray analysis. The microarray analysis was used to detect the differential gene expression of mouse brain in acute repetitive hypoxia conditions. Multiple bioinformatics methods were used to reveal the molecular mechanism associated with HPC.

## RESULTS

### Mouse model of HPC

The mice exposed to hypoxic condition for six times (H6) were adopted as the HPC animal model. The mice of repeated six times sham-HPC were employed as the control group. The tolerance duration of the HPC mice were recorded when the asthmoid respiration of hypoxic mice just appeared. It was noted that the tolerance duration of mice to hypoxia was significantly prolonged as the number of exposure increased. The mean tolerance time of H6 mice was up to 6.8 times longer than that of the mice on their first exposure to hypoxia even though the tolerance times had some animal differences [7, 8]. A linear relationship between the tolerance times and the number of times exposed was observed in the HPC mice (y = 16.471x + 0.888, *R*^2^ = 0.9882), suggesting a positive correlation between the exposure times and the tolerance duration to some extent. But there was some individual variance in the HPC mice at the different hypoxic exposure time owing the biological differences.

### Screening and analysis of differential gene

**Figure 1:**
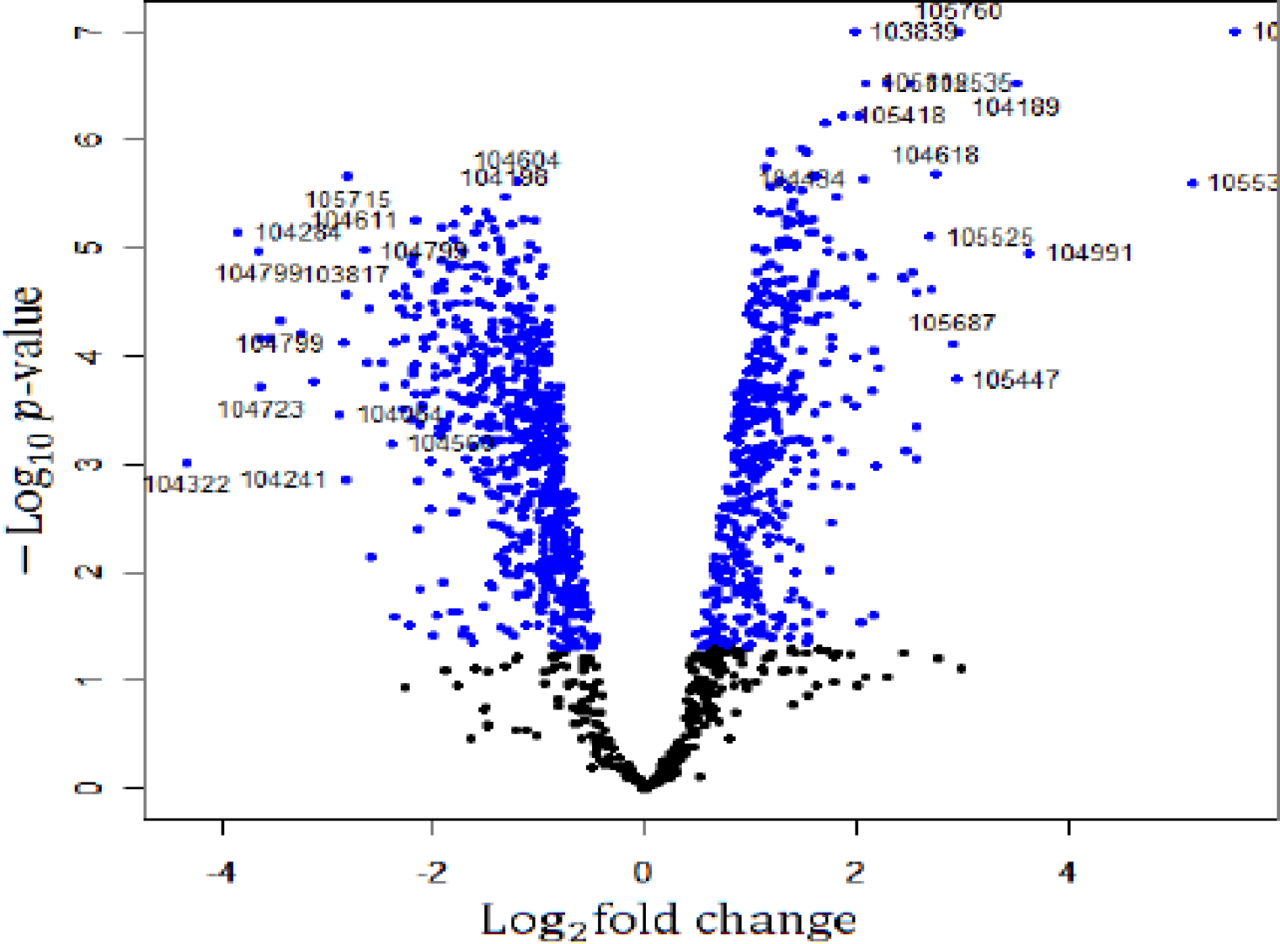
A volcano plot of the genes in microarray The Log^2^ fold changes and their corresponding –log^10^ *p*-value of all genes were taken for construction of the volcano plot in the microarray. The genes with *p* < 0.05 are depicted in blue dots. All other genes that were not found to be significant altered are in black dots in this array.

The gene expression profiles in the H6 mice and the sham-HPC mice were examined using an Affymetrix microarray. The random variance model (RVM) *t*-test was applied to screen the differentially expressed genes from these two groups, which was suitable for cases of small samples [13, 14]. Based on the significant analysis and the false discovery rate (FDR), the differentially expressed genes were selected at a cutoff threshold of *p* < 0.05 and FDR < 0.05. Compared with the sham-HPC mice, a total of 1175 genes of H6 mice showed significant difference in their expression levels. Among these genes, 460 genes were up-regulated in the H6 mice with a maximum fold change of 47.26 times. 715 genes were down-regulated genes in the H6 mice with the fold change of 20 times. A volcano plot of all the genes in the microarray is shown in Figure 1. The top 20 differentially expressed genes in the H6 mice are listed in Table 1.

**Table 1:** The top 20 differentially expressed genes in HPC mice

Gene Symbol	*p*-value	FDR value	Geom mean of intensities in experimental group	Geom mean of intensities in control group	Fold change	Trend
*Glra1*	8.157E-12	1.813E-11	2650.31	56.08	47.26	up
*Foxf1a*	3.199E-11	3.555E-11	551.13	70.9	7.77	up
*Nefh*	6.781E-11	5.023E-11	1345.14	343.73	3.91	up
*Tppp3*	0.0000003	4.896E-05	1202.14	286.92	4.19	up
*Ankrd55*	0.0000003	4.896E-05	368.15	76.36	4.82	up
*Slc17a6*	0.0000003	4.896E-05	976.2	174.12	5.61	up
*Sncg*	0.0000003	4.896E-05	984.48	87.66	11.23	up
*Vamp1*	0.0000006	7.63E-05	3440.73	956.74	3.6	up
*Foxf1a*	0.0000006	7.63E-05	380.63	94.12	4.04	up
*Adamts9*	0.0000012	0.0001141	156.57	56.26	2.78	up
*Dennd3*	0.0000013	0.0001141	155.66	69.33	2.25	up
*Apold1*	0.0000013	0.0001141	239.55	84.38	2.84	up
*Dock5*	0.0000018	0.0001296	180.49	82.61	2.18	up
*Prune2*	0.0000021	0.0001296	205.25	30.72	6.68	up
*Asah2*	0.0000022	0.0001296	470.21	158.82	2.96	up
*Cd38*	0.0000022	0.0001296	400.77	131.11	3.06	up
*Cdkn1a*	0.0000023	0.0001296	910.32	219.95	4.14	up
*Spnb3*	0.0000024	0.0001296	341.48	785.22	0.43	down
*Ahnak2*	0.0000024	0.0001296	201.92	84.16	2.4	up
*Slc6a5*	0.0000025	0.0001296	2625.3	74.1	35.43	up

### GO analysis based on differential genes

The Fisher’s exact test and χ^2^ test were used to classify the GO categories, and the FDR was used to calculate and adjust the *p*-value [15]. A low FDR indicates that the error is small in judging the *p*-value. The *p*-value was computed for the GO categories for the differential genes. Enrichment was used to measure the significance of assigned GO functions. The analysis revealed 113 up-regulated and 138 down-regulated functions that were significantly enriched among the differentially expressed genes (see Supplementary Table 1 and Supplementary Table 2). The significantly enriched functions of up-regulated genes included ion transport, neurotransmitter transport, synaptic transmission, signal transduction, and cranial nerve development. The significantly enriched functions of down-regulated genes included calcium and potassium ion transport, nervous system and brain development, and neuron migration and neuronal differentiation.

**Figure 2:**
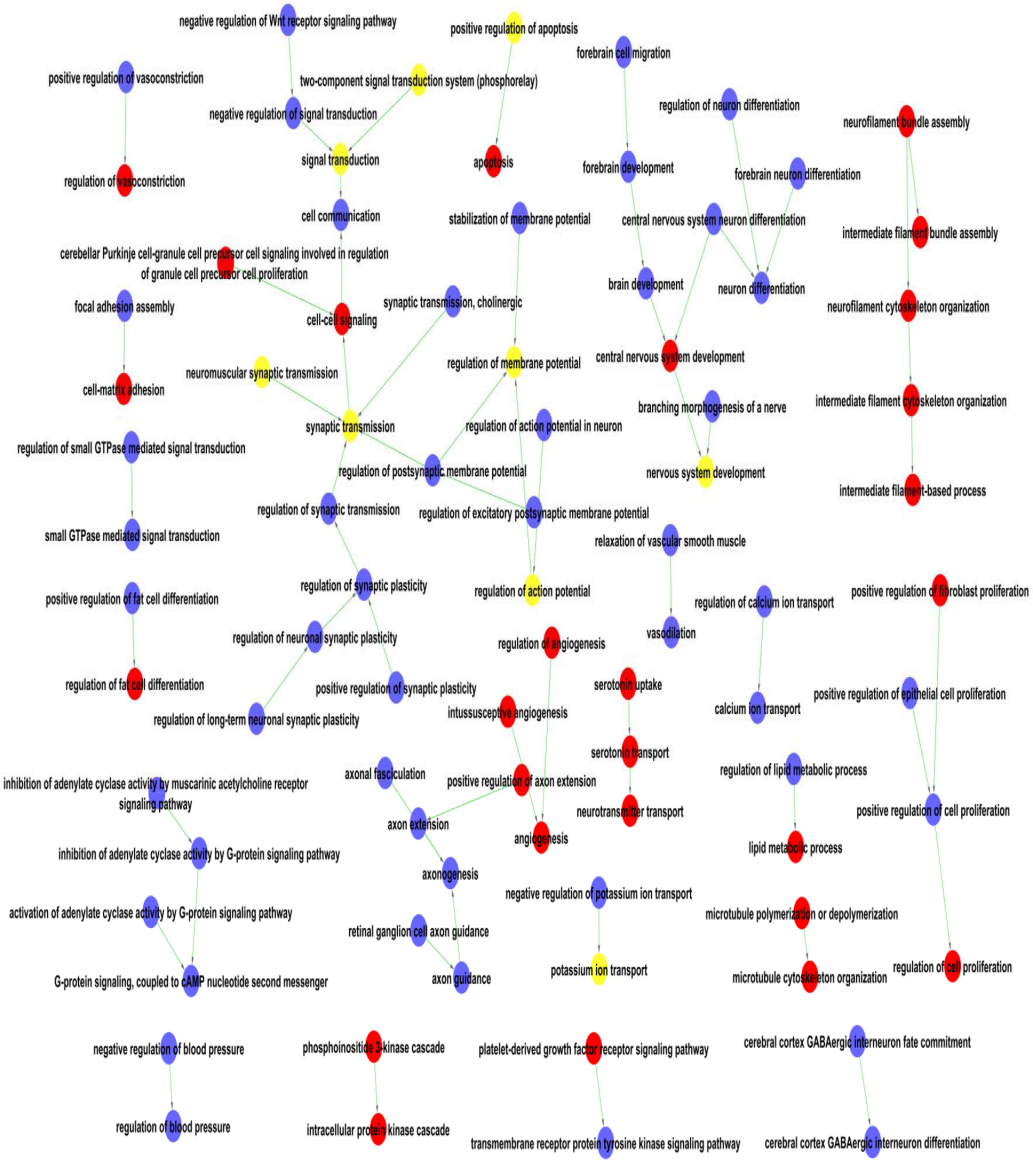
The function networks of the differentially expressed genes by GO terms One node represents one GO term and function of one differential gene. The red nodes represent the up-regulated genes, the blue nodes represent the down-regulated gene and the yellow nodes mean both up and down regulated gene. The lines represent the relationship of the functions, with arrow from the lower ranking Go term pointing towards to the higher one.

All the significantly enriched GO terms were assembled into a GO map (see Figure 2) to help to visualize the interactive networks. The GO map can systemically construct the interaction networks of significant GO terms [13]. The nodes in the networks represent the functions of differential genes, and the lines represent the relationship between the functions. Twenty-one sub-networks were revealed including signal transduction, synaptic transmission, brain development, neuron differentiation, axonogenesis and cell proliferation. The interaction networks in the GO map suggested that HPC may trigger a series of signal transduction events involving GTPase-mediated signal transduction and G-protein signaling pathways. The cell proliferation, intermediating filament, and neurotransmitter transport functions were up-regulated in the H6 mice brains, while the vasodilatation, calcium ion transport, and blood pressure functions were down-regulated in the H6 brains.

### Pathway analysis based on the significant differential genes

**Figure 3:**
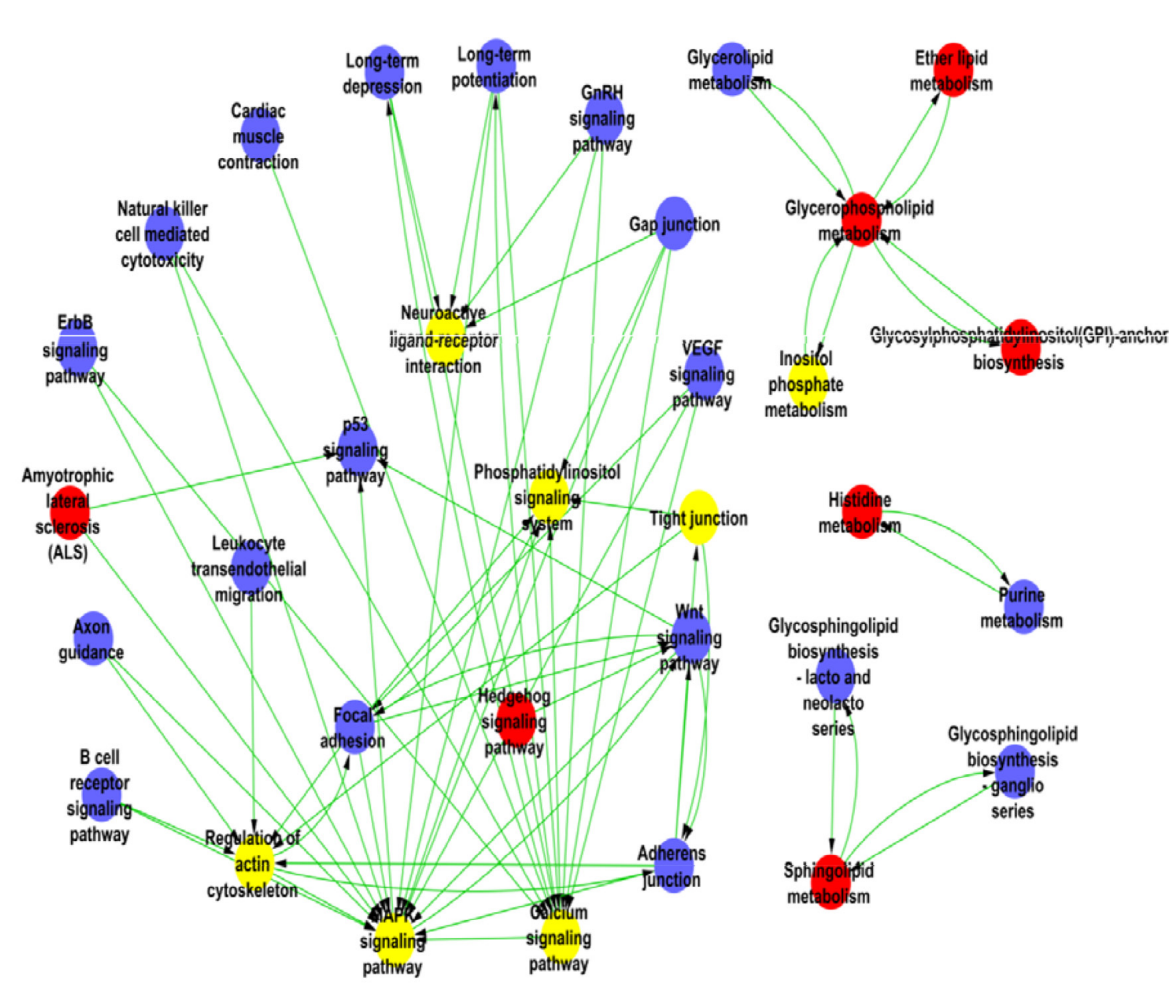
The pathway network of the differentially expressed genes in HPC mice The nodes represent the pathways and the lines represent the relationship between the pathways. The red nodes mean up-regulated pathways; the blue nodes mean down-regulated pathways and the yellow mean both up and down regulated pathways.

The main pathways linked to the significant differential genes were assigned based on the pathway maps of KEGG, Biocarta and Reactome. Nineteen of the up-regulated signaling pathways are listed in Supplementary Table 3, including neuro-active ligand-receptor interaction, mitogen-activated protein kinase (MAPK) signaling pathway, calcium signaling pathway, inositol phosphate and ether lipid metabolic pathways [15]. The relationships among these pathways were built using the PathNet, summarized in Figure 3. The Path-Network was the interactive network of significant pathways from the differential expression genes, which was constructed according to the interaction pathways of KEGG database. In these pathway networks, MAPK signaling pathway had the greatest degree; it existed at the central position of Path-Network by mediating the cascade reactions. MAPK is the serine-threonine kinase that regulates multiple cellular functions such as mitogens, inflammatory cytokines, gene expression and cell survival or apoptosis, etc. MAPK is also a key component for extra-cellular stimuli. During HPC process, the multiple molecular reactions in cells could be initiated by activating MAPK pathway to prevent hypoxia damage [16].

### Signal network analysis based on differential genes

**Figure 4:**
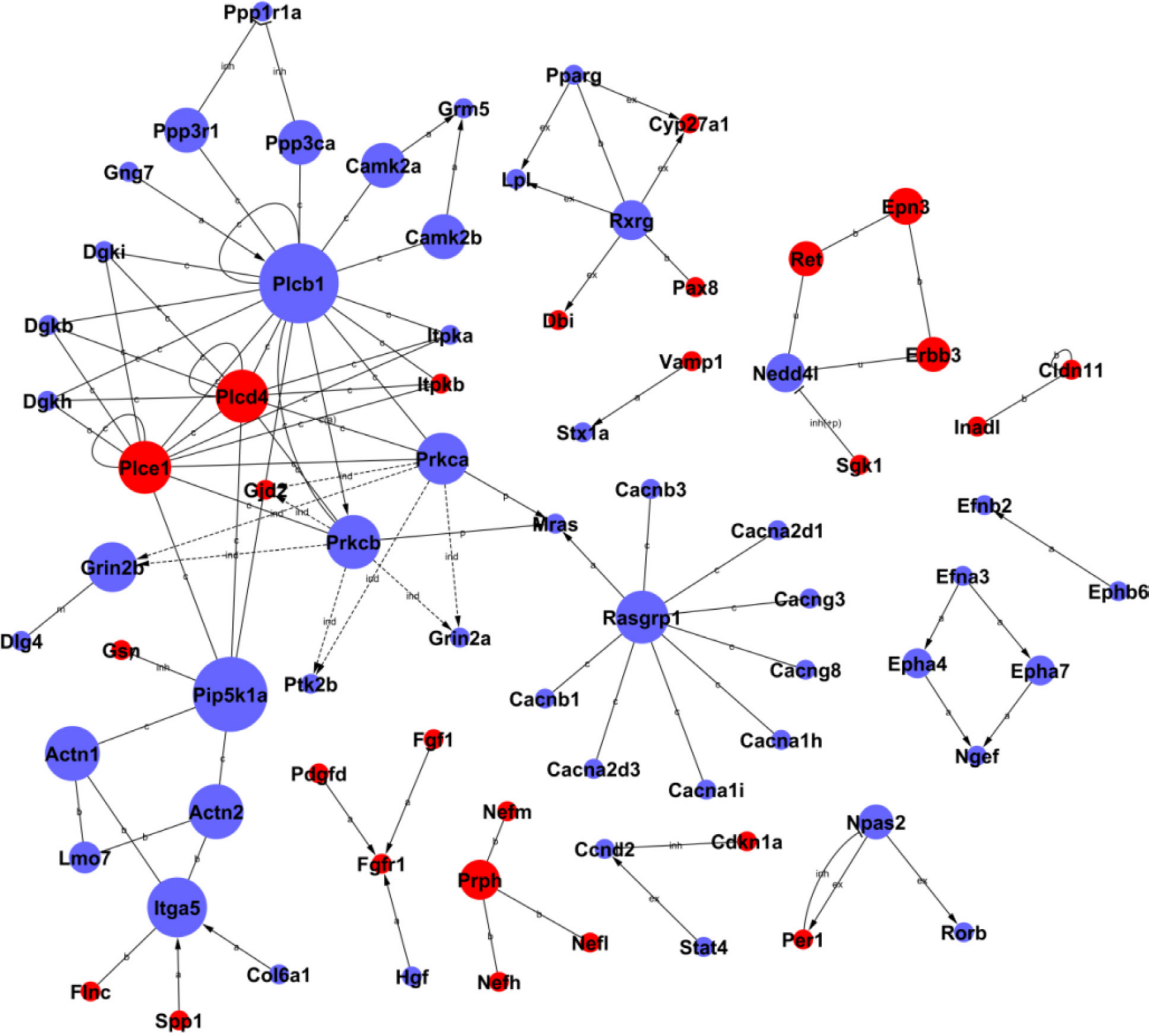
The interaction networks of differentially expressed genes in HPC mice The nodes represent the genes, the red nodes mean the up-regulated genes and the blue nodes mean the down-regulated genes. The edges indicate the interaction between the genes. The arrows show the action direction. The size of nodes means the level of expressed degree. All the nodes were marked with k-core values. The genes with higher k-core values are more centralized in the network and have a stronger capacity of modulating adjacent genes.

Signal networks of gene interactions were constructed to reveal the relationships among the significant differential genes, and to map the upstream and downstream of target genes (see Figure 4). When there was evidence that two genes interacted with each other, an interaction edge was assigned between the two genes. To investigate the entire signal networks, the most important nodes were identified computationally. The connectivity was defined to measure how closely a target gene correlated with other genes in this network. The degree was estimated according to the number of genes correlated with the target gene. The characteristics of these genes were described by the betweenness centrality of nodes, which reflected the importance of a node in a graph relative to other nodes. It was noted that *Plcb1,* encoded phospholipase that had crucial roles in lipid metabolism, was significantly down-regulated in the H6 mice. The interaction networks of differential genes demonstrated that *Plcb1* displayed the largest betweenness centrality, implying that this gene was mostly involved in interactions with other genes in HPC (showed in Table 2). *Plcb1* showed the highest activity and might perform as an intermediary in the networks, suggesting that *Plcb1* was a hub gene and a key node in these signal networks.

**Table 2:** The interaction networks of hub genes in HPC mice brain

Gene symbol	Description	Betweenness centrality	Degree	Indegree	Outdegree	Style
*Plcb1*	Mus musculus phospholipase C, beta 1 (Plcb1), transcript variant 2, mRNA.	0.996131	17	16	16	down
*Pip5k1a*	Mus musculus phosphatidylinositol-4-phosphate 5-kinase, type 1 alpha (Pip5k1a), mRNA.	0.7864583	6	5	6	down
*Itga5*	Mus musculus integrin alpha 5 (fibronectin receptor alpha) (Itga5), mRNA.	0.3208333	5	5	3	down
*Actn1*	Mus musculus actinin, alpha 1 (Actn1), mRNA.	0.271875	3	3	3	down
*Actn2*	Mus musculus actinin alpha 2 (Actn2), mRNA.	0.271875	3	3	3	down
*Prkcb*	Mus musculus protein kinase C, beta (Prkcb), mRNA.	0.2330357	9	4	8	down
*Plcd4*	Mus musculus phospholipase C, delta 4 (Plcd4), transcript variant 1, mRNA.	0.219122	11	11	11	up
*Plce1*	Mus musculus phospholipase C, epsilon 1 (Plce1), mRNA.	0.219122	11	11	11	up
*Rasgrp1*	Mus musculus RAS guanyl releasing protein 1 (Rasgrp1), mRNA.	0.2	9	8	9	down
*Prkca*	Mus musculus protein kinase C, alpha (Prkca), mRNA.	0.1607143	8	3	8	down

### Gene co-expression networks based on GO and pathway analyses

On basis of normalized signal intensities for these differential expression genes, the co-expression networks were constructed with an aim to identify the core regulatory genes [17, 18]. The degree centrality, as a simplest and most important index, was defined to describe the number of links that one node connecting to the other in the network. In addition, the k-cores were introduced as a method to simplify the analysis of graph topology. The k-core presented as a sub-network where the nodes were connected to others.

**Figure 5:**
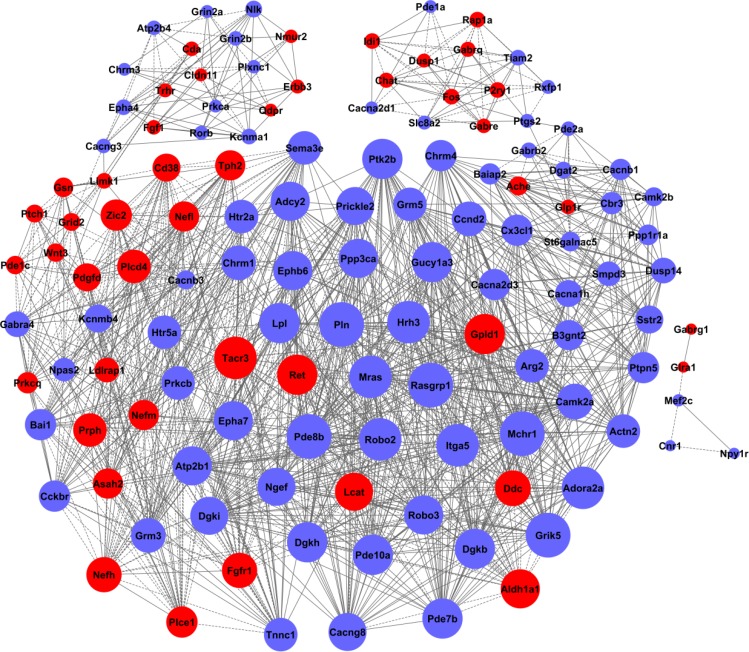
The co-expression network of genes formed from the control group The genes contained in the significant GO terms were analyzed and identified by the gene co-expression network with the k-core algorithm. The node represents the gene, the red nodes mean the up-regulated genes, the blue nodes mean the down-regulated, and all the nodes were marked with the k-core values. The genes with higher k-core values are more centralized in the network and have a stronger capacity of modulating adjacent genes. The line represents the regulatory relationship, the solid line means the positive regulation and the dotted line means the negative regulation.

**Figure 6:**
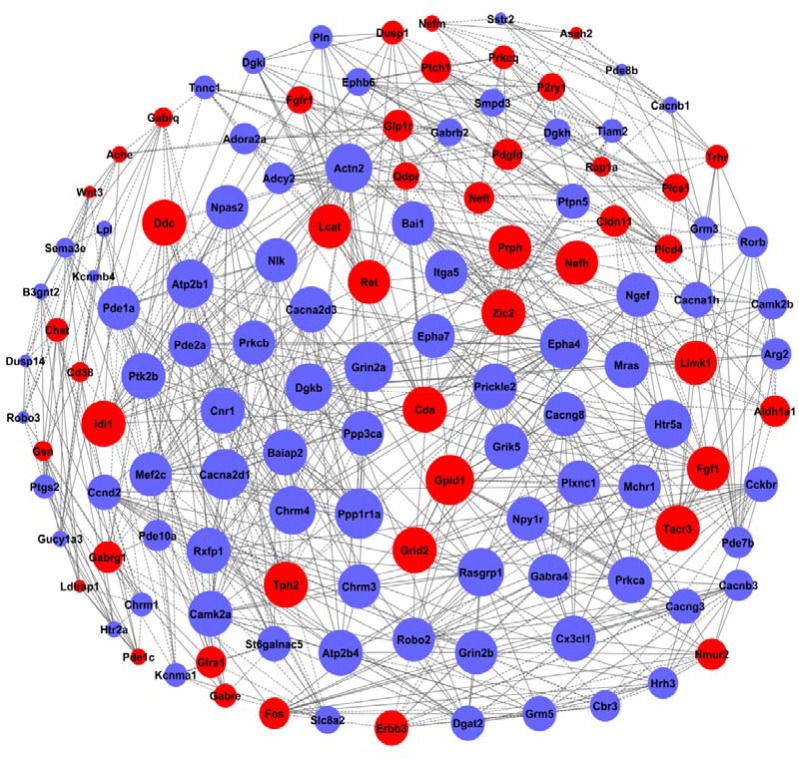
The co-expression network of genes formed from the HPC experimental group The genes contained in significant GO terms were analyzed and identified by the gene co-expression network with the k-core algorithm. The node represents the gene, the red nodes mean the up-regulated genes, the blue nodes mean the down-regulated, and all the nodes were marked with the k-core values. Genes with higher k-core values are more centralized in the network and have a stronger capacity of modulating adjacent genes. The line represents the regulatory relationship, the solid line means the positive regulation and the dotted line means the negative regulation.

The gene co-expression networks for the H6 and sham-HPC mice were also constructed based on the gene expression data (seen in Figure 5 and Figure 6). The dominated genes were screened and identified based on the co-expression network and status changes. The co-expression changes of these key genes reflected the differences of brain samples between the H6 mice and sham-HPC mice.

### Amplification and verification of differential expression genes by RT-PCR

**Figure 7:**
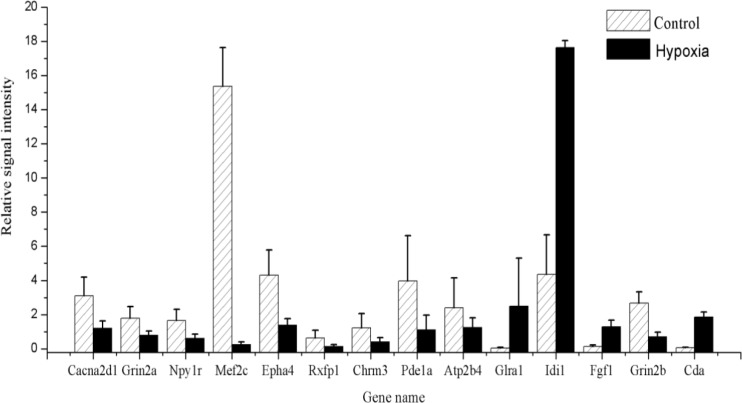
The relative signal intensity of genes between the control and HPC group

Quantitative real-time RT-PCR was used to validate the reliability of microarray results. Fourteen hub genes with more than 2-fold differential expression in the networks were studied. The gene expression levels were quantified relative to the expression level of β-actin by the 2^-ΔΔCt^ method [19]. The RT-PCR results suggested that ten differentially expressed genes (*Cacna2d1*, *Grin2a*, *Npy1r*, *Mef2c*, *Epha4*, *Rxfp1*, *Chrm3*, *Pde1a*, *Atp2b4* and *Grin2b*) were significantly down-regulated in the H6 mice brains compared with those in the sham-HPC mice. Four genes (*Glra1*, *Idi1*, *Fgf1* and *Cda*) were significantly up-regulated in the H6 mice (shown in Figure 7). The findings from the RT-PCR experiments, including the variation trends and expression levels of these fourteen genes, were in line with that from the microarray analysis.

Based on our data, the gene networks were constructed for the major genes related to HPC mice. The results showed that: (1) there were marked differences in the gene expression profiles between the HPC mice and control group; (2) the hub genes such as *Plcb1* was crucial in HPC process; and (3) the microarray and gene network analyses were very powerful tools to identify the HPC hub gene.

## DISCUSSION

The ability to sense oxygen levels and maintain oxygen homeostasis is crucial for cell survival. The hypoxic-sensitive regulation of gene expression for oxygen status can be converted into appropriate cellular responses. Although there are some core transcriptional pathways in cells, the signaling cascades can be modified to allow the diversity and specificity in transcriptional output. Pathophysiological conditions and diseases such as cardiovascular disease, stroke and cancer often lead to a significant decrease in oxygen supply to cells. Hypoxia can also induce the significant metabolic changes. Oxygen homeostasis in organisms is a complex process of integration and regulation via the hub gene and protein expression. Successful adaptation to hypoxia involves changes in the gene programs that modulate cellular metabolism for substances and energy [20]. Thus, the gene networks that govern oxygen homeostasis are of great significance for investigation into the molecular mechanisms of hypoxia and HPC. The microarray and gene network analyses are effective approaches to identify HPC-related genes and reveal the molecular mechanisms of HPC [21]. Generally, high-throughput data have the features of high variability, low reproducibility and non-specific noise. The subgroups of genes within the dataset may be associated with a generalized response to a given stimulus [22]. This effect can be ameliorated using comprehensive bioinformatics approaches including gene analysis, GO analysis, gene network and pathway analysis to enrich the relevant and responsive genes for HPC.

In our study, the oxygen content of mice exposed to hypoxia was determined via the pHOxCO-Oximeter methods [5]. The results indicated a marked relationship between the number of hypoxia exposures and the remained oxygen concentration or oxygen content. The values of oxygen content, oxygen saturation and oxyhemoglobin were markedly decreased in the HPC groups. Significant differences were evident between these groups (*p* < 0.05 or *p* < 0.01), indicating that the oxygen content was markedly decreased in successive HPC groups. Our data indicated that the HPC model was successful and the brain samples could be used to the hypoxic experiments [7, 8].

The approaches provide a comprehensive overview for thousands of HPC related genes, and enormously expand our view of molecular and metabolic changes in hypoxia and HPC responses. The two most important gene discovery techniques available to gene hunter are the DNA microarray screening and cDNA library screening. We have used them to successfully analyze the diverse animal models of hypoxia and HPC. To comprehensively understand the processes of HPC in mice, the whole brain was subjected to HPC to identify the differentially expressed genes. In our study, the differentially expressed genes in HPC brains were found to be involved in ion transport, neurotransmitter transport, synapse transduction, neuropeptide signal conduction, and cranial nerve development. Bioinformatics combined with gene network analysis has been effectively used to identify the hub genes that may be as therapeutic targets of hypoxia related diseases [21].

In our study, 1175 HPC-related differentially expressed genes have been identified. By filtering significant differential genes and building co-expression networks, a variety of methods are available to provide a more systemic and intuitive insight into the HPC process [23]. Of the 1175 differentially expressed genes, 113 were up-regulated and 138 were down-regulated in the HPC mice brains compared with those the sham-HPC controls (Supplementary Table 1 and Supplementary Table 2). The GO analysis of 1175 differentially expressed genes suggested that the cellular signaling processes, especially, the electron transport rate of the respiratory chains, were the most important. The GO terms for the response of brain cells to hypoxic stimuli were confirmed by the GO map analysis. The high correlation between the HPC and the constructed gene networks reflected the high sensitivity of differentially expressed genes *Plcb1*, *Cacna2d1*, *Atp2b4*, *Grin2a*, *Grin2b*, and *Glra1* to HPC. Meantime, the pathway network showed that these genes also participated in hub pathways. Combined the results of pathway network and gene co-expression network (see Supplementary Table 3 and Supplementary Table 4), we defined *Plcb1*, *Cacna2d1*, *Atp2b4*, *Grin2a*, *Grin2b* and *Glra1* as the main hub genes tightly associated with HPC.

Our results indicated that *Plcb1* had the largest betweenness centrality and more interactions with other differential genes, suggesting that *Plcb1* is a main key gene in the HPC networks. *Plcb1* participated in eleven significant down-regulated pathways, including calcium signaling pathway, Wnt signaling pathway, vascular smooth muscle contraction and so forth. *Plcb1* encodes a phospholipase that belongs to the PLC family [24]. The main function of Plcb1 is to produce the second messengers such as inositol triphosphate (IP_3_) and diacylglycerol (DAG). After binding to the receptors on endoplasmic reticulum, IP_3_ can open calcium ion channels in the membrane, which allows calcium ions to cross into the cytoplasm [25]. *Plcb1* was markedly down-regulated in the H6 mice brains, indicating that calcium levels in the cytoplasm may decline to avoid calcium overload and prevent hypoxia damage. Interestingly, the isogeny both *Plcd4* and *Plce1* were markedly up-regulated in the H6 mice, inferring that these two genes also play an important role in HPC. In addition, *Pip5k1a* encoded phosphatidylinositol-4-phosphate 5-kinase type 1 alpha, markedly down-regulated and had the larger betweenness centrality and interactions, suggesting that *Pip5k1a* was a key gene in the HPC networks.

*Cacna2d1* and *Cacna2d3* encoded the subunits α2δ1 and α2δ3, is a protein complex in voltage-dependent L-type calcium channel that maintains intracellular calcium level [26, 27]. *Cacna2d1* was down-regulated by at least two fold in the HPC mice brains. The pathway analyses indicated that *Cacna2d1* was responsible for ion transport in the MAPK pathway. MAPK is a serine-threonine kinase that regulates multiple cellular functions such as mitogens, proinflammatory cytokines, gene expression, and cell survival or apoptosis. Further, MAPK is a major component in a series of cascade reactions and an important factor in extra-cellular stimuli. During the HPC, these reactions in the cells could be initiated by activating the MAPK pathway to prevent hypoxia damage [28]. In addition, the co-expression network showed that the expression levels of *Cacna2d1* had markedly difference between the H6 mice and sham-HPC mice. In the sham-HPC mice, *Cacna2d1* expression was positively correlated with the expression of the gene *Dusp1*, *Gabre* and *Fos*, and negatively correlated with *Idil*. While in the H6 mice, *Cacna2d1* expression was positively correlated with the gene *Atp2b4*, *Ppp1r1a*, *Cacna2d3* and *Grin2a*, and negatively correlated with *Tph2*. The co-expression results indicated that the expression levels of both *Cacna2d1* and *Cacna2d3* decreased in the HPC. These findings suggested that inhibition of calcium influx by down-regulation of *Cacna2d1* may be one of the protection mechanisms of HPC.

The gene *Atp2b4*, encoded a membrane calcium transporter, i.e., adenosine triphosphatase (ATPase) [29], was down-regulated 2.1 times and was co-expressed with *Cacna2d1* in the H6 mice brains. The GO analysis showed that the main function of *Atp24* was to transport calcium. The pathway analysis indicated that *Atp2b4* also participated in the calcium signaling pathway. The protein encoded by *Atp2b4* belongs to the family of P-type primary ion transport ATPases. The main function of the enzyme is to remove the bivalent calcium ions from eukaryotic cell against high concentration gradients, and plays a crucial role in maintaining intracellular calcium homeostasis. The ATPase uses an energy-consuming process to promote calcium outflow or storage in organelles, resulted in decline of intracellular calcium [30]. The down-regulation of *Atp2b4* in HPC mice may lead to a decrease in the ATPase in cell membrane, implying that calcium may be transported by low energy-consuming processes related to *Cacna2d1* and *Cacna2d3*. Thus, the HPC tissues could use an endogenous mechanism to reduce the energy consumption and contribute to the protective effect of HPC.

*Grin2a* and *Grin2b* were also significant differential genes that had markedly different co-expression levels between the HPC and the control group [31]. The pathway network showed that these two genes involved in calcium signaling pathway, neuroactive ligand-receptor interaction and long-term potentiation. *Grin2a* and *Grin2b* encoded the ionotropic glutamate receptor N-methyl-D-aspartate NMDA2A and NMDA2B subtypes, respectively. This receptor was a member of ionotropic glutamate receptor and involved in long-term potentiation. This receptor was linked to the ion channel that Mg^2+^ can block Ca^2+^ influx into the cell via the NMDA channel in resting state. When the NMDA receptors activated, the membrane channels open, Mg^2+^ released from the cell and Ca^2+^ influxes into the cell, resulting in Ca^2+^ overload and cellular damage. The co-expression network results showed that *Grin2a* and *Grin2b* were both down-regulated in the HPC mice, suggesting that the levels of NMDA2A and NMDA2B may decrease in the HPC mice. Our study indicated that the level of glutamate in the HPC brain markedly reduced, inferring that HPC could protect the cell from hypoxic damage via reducing calcium overload. This key process can be suppressed by blocking the NMDA receptor subtype and reducing glutamate level.

*Glra1* mainly encoded the α_1_ subunit of a glycine receptor that consisted of three α subunits and two β subunits [32]. The glycine receptor was a member of cys-loop family of ligand-gated ion channels, which was responsible for mediating the inhibitory effects of glycine. They widely distributed in central nervous system and were important inhibitory receptors. *Glra1* was up-regulated at least 47-fold in the HPC mice, the biggest fold change among the up-regulated genes. The GO analysis showed that the functions of *Glra1* included the ion transport, synaptic transmission, neuropeptide signaling pathway, chloride transport, membrane potential and action potential. The pathway analysis indicated that *Glra1* may play a role in neuroactive ligand-receptor interaction. The high levels of glycine could exert neuroprotective effects by activating the glycine receptor and increasing the NMDA subunit component [33]. The evidence suggested that glycine could protect cells from hypoxia damage via up-regulating the α_1_ subunit and inhibiting the activation of membrane voltage-dependent Ca^2+^ channels to block Ca^2+^ influx into the cell.

All eukaryotic cells require oxygen for oxidative phosphorylation to generate high-energy phosphates adenosine triphosphate (ATP). The energy-rich molecules are used to maintain the normal cellular functions such as proliferation, membrane transport and metbolic reactions [34]. Significant changes in the metabolic responses as a result of adaptation to hypoxia can be observed in the cells of HPC animals. More importantly, the metabolic rate depression is achieved by targeting and coordinating the rates of energy-producing pathways and energy-utilizing functions, and reorganizing the priorities for ATP expenditure [35]. Currently, some novel biomarkers and metabolic pathways in HPC are being investigated using modern analytical technologies to obtain global omic profiles in the cell/organ of interests in our laboratory. The modern omics allow us to make major advances in understanding of how organisms adapt their metabolism to endure hypoxia. Especially, the “global view” is critical in showing both the breadth of cellular responses and commonalities of molecular mechanisms that underlie organismal adaptation to hypoxia. In previous work, we have seen some crucial changes in corresponding endogenous metabolites such as peptides, amino acids, sphingolipids, and spermidine, etc, and these metabolites had important roles in providing ATP and maintaining cell survival. Some of the metabolites had been identified with HPLC-HRMS and the standard compounds [8]. The functions and pathways of these metabolites need to further study.

The gene expression profiles in brain of HPC mice were generated and integrated using a multi-step bioinformatics strategy to determine the HPC-related hub genes, pathways, network and biological processes. Our results indicated that the expression profiles of the key genes in the HPC mice were markedly different from that in the sham-HPC mice. Several critical hub genes, as the key members of significant pathways or gene networks, were identified by this comprehensive analysis. Combined the results of pathway network and gene co-expression network, (see Supplementary Table 3 and Supplementary Table 4), we defined *Plcb1*, *Cacna2d1*, *Atp2b4*, *Grin2a*, *Grin2b* and *Glra1* as the main hub genes tightly related with HPC. The results showed that the up-regulated genes were mainly involved in the ion transport, neurotransmitter transport, synapse transduction, signal conduction, cranial nerve development and neuropeptide signal path, while the down-regulated genes were mainly involved in the calcium ion transport, nerve phylogenies, brain development, neuron migration and neuron differentiation. During the HPC, the cells may use several molecular mechanisms to change the metabolic pathways, respiratory chain and electron transport for decreasing the energy requirement and intracellular Ca^2+^ level to prevent calcium overload and protect the organism from hypoxia damage. To summarize, we firstly found that the global gene expression profiles in mice brain tissues were significantly different between the HPC and the sham-HPC control mice. The findings of the hub genes and integrated gene networks may provide pivotal and useful information for exploring the molecular mechanisms in HPC, cerebral ischemic injury and neuroprotection.

## MATERIALS AND METHODS

### Drug and reagents

RNA extract kit included Qiagen Rneasy Mini Kit (number 217004, Qiagen Co., Ltd., Germany), Qiagen Rnase-free Dnase I (number 79254, Qiagen Co., Ltd., Germany), Ambion WT Expression Kit (Ambion Co., Ltd., USA), Affymetrix gragmentation and labelling kit (Gene Chip 2 WT Terminal Labeling Kit and Controls Kit 30 times, Affymetrix Co., Ltd., USA). Affymetrix reagents were the eneChip2 Hybridization, Wash and Stain Kit (Affymetrix Co., Ltd., USA).

### Animals and HPC experiments

Adult male Imprinting Control Region (ICR) mice, body weight 18–20 g, were obtained from the Laboratory Animals Center of Capital Medical University. The experiments were carried out in accordance with the current guidelines for the care of laboratory animals and ethical guidelines for investigations of experiments in conscious animals [36, 37]. In addition, the experimental protocols employed were approved by the Animal Care and Use Committee of Capital Medical University and consistent with the NIH Guide for the Care and Use of Laboratory Animals (NIH Publications No. 80-23). The six mice were allocated randomly to the HPC group and the sham-HPC group as the control. A “power test” based on the standard deviation of the mean have been carried out to determine the number of animals required. All mice were bred in isolated cages.

Acute repetitive hypoxia models were constructed at room temperature (20 ± 1°C), and used to identify the main differentially expressed genes related to HPC. The acute repetitive hypoxia mice were treated as reported previously [5–8, 38]. Briefly, a mouse was placed in a 125 ml airtight jar containing air and the bottom of jar with Ca(OH)_2_ to absorb CO_2_. The jar was immediately sealed with a rubber stopper and smeared with Vaseline. Simultaneously, the tolerance time and behavior of the mouse were observed. The mouse was taken out of the jar as soon as the asthmoid respiration just appeared. This is the first time of hypoxic preconditioning treatment. After ten minutes recovery under air condition, the mouse was moved to another new jar with same volume fresh air and the jar was hermetically sealed with rubber plug immediately, and to duplicate a progressive hypoxia environment. The control mice placed in open jars for the same amount of time were used as the normoxic sham-HPC control group. These mice had just transferred to another open jar with same volume fresh air, but did not be sealed with rubber plug. The procedure was performed repeated six times (H6) for the experimental HPC mice and sham-HPC control mice [7]. After the experiment the animals were decapitated, and their whole brain tissues were collected on ice and saved in liquid nitrogen for use.

### Microarray experiment

The procedures of microarray experiments mainly included: isolation and validation of the total RNA from the mouse brain, the reverse transcription and *in vitro* transcription of RNA (i.e. the first and second strand synthesis of first-cDNA, synthesis and purification of cRNA, synthesis and purification of 2nd-cDNA), and then label and hybridization of the samples (including gragmentation of 2nd-cDNA, label of gragmentated 2^nd^-cDNA, chip hybridization, and scrubbing and scanning of array) [39–41].

For the Affymetrix microarray profiling, total RNA was isolated with TRIzol reagent (Invitrogen, Canada) from whole brain and purified using a RNeasy Mini Kit (Qiagen, German), including a DNase digestion treatment. The RNA concentration was determined by absorbance at 260 nm. The quality control standards of A260/A280 were 1.8–2.1 using the Nano Drop 2000 (Thermo, America). The cDNA from the repeated six times HPC group and the sham-HPC control group was hybridized to Mouse Gene 1.0 ST GeneChip arrays (Affymetrix, USA) according to the manufacturer’s instructions. The Affymetrix Expression Console software (version 1.2.1) was used for microarray analysis. Raw data (CEL files) were normalized at transcription level using the robust multi-average method (RMA workflow). The median summarization of transcript expression levels was calculated. The gene-level data were then filtered to include only the probe sets in the ‘core’ meta-probe list, which represents the RefSeq genes [40].

For each sample, three biological replicates were performed. The Two ClassDif was used to screen and analyze the differentially expressed genes in these samples, which was the *t*-test method via an adjustment of model for random variance. This method effectively improves the accuracy of characteristic screening and reduce the false positive rate. All arrays were washed, stained, and read by a GeneChip Scanner 3000 (Affymetrix). The fluorescence signal was excited at 570 nm, and the data were collected on a confocal scanner at 3 lm resolution. The data were analyzed by the GeneChip Operating Software 1.4.

### Differential gene analysis

Significant differential genes between the experimental group and control group were screened using the relative variance method (RVM) from the *t*-test [41]. The threshold of truly significant genes was taken to be *p*-value < 0.05 and the false discovery rate (FDR) < 0.05.

### Gene ontology and pathway analysis

Gene Ontology (GO) analysis was applied to analyze the main functions of the differentially expressed genes [42]. GO annotations are the main functional classification used by the National Center for Biotechnology Information (NCBI). A total of 1175 genes in the significant differential gene group were classified and analyzed based on their GO functional annotations. The GO terms assigned to these genes were obtained and examined using the Fisher’s exact test and chi-square (χ^2^) test to calculate the level of significance. The threshold was taken to be *p*-value < 0.05 and the FDR value < 0.05. For the functions in significant category, the enrichment (*R*_e_) was calculated as *R*_e_ = (n_f_/n)/(N_f_/N), where n_f_ is the number of differential genes within a GO category of interest, n is the total number of genes in the category, N_f_ is the number of differential genes in the entire microarray, and N is the total number of genes in the microarray.

Pathway analysis of significant differential genes was based on the KEGG (Kyoto Encyclopedia of Genes and Genomes), BioCarta and Reactome databases [42]. The Fisher’s exact test and χ^2^ test were used to classify the enrichment of pathway categories and the threshold of significance was defined by *p*-value < 0.05) and the FDR value < 0.05. For the pathways in the significant category, the enrichment (*R*_e_) was calculated as *R*_e_ = (i/m)/(k/N), where the i is the number of differential genes within a pathway category of interest, m is the total number of genes in that category, k is the number of differential genes in the entire microarray, and N is the total number of genes detected in the microarray.

The PathNet program was constructed based on the interactions among the pathways of KEGG database to find the interactions among significant pathways directly and systemically [12]. Here, we used the PathNet R package to build an interaction network of significant pathways from the differential expression genes to identify differential networks that might help explain why certain pathways were activated in HPC.

### Gene co-expression network analysis

The signal intensities of differentially expressed genes were used to build the gene co-expression networks [43]. Pearson’s correlations (*R*) for each pair of genes were calculated and used to select the significant correlation gene pairs. A gene-gene interaction network was established according to the correlations between the genes. The network nodes represent the genes, and the edges between the genes indicate the interaction between these genes. All the nodes were marked with degrees, defined as the number of links that one node with the all the other nodes in the network. The genes with the higher degrees occupied more of the central positions in the network and had a stronger capacity to modulate adjacent genes than the genes with the lower degrees. In addition, the k-core model was applied to describe the characteristics of the network including the centrality of genes within the network and the complexity of the sub-networks. Based on the relationships between the genes, they were divided into several sub-networks and marked red (up-regulated) or blue (down-regulated).

### RT-PCR confirmation of differentially expressed genes

Real-time RT-PCR was conducted to confirm the differential expression of 14 selected key genes, namely, *Cacna2d1*, *Grin2a*, *Npy1r*, *Mef2c*, *Epha4*, *Rxfp1*, *Chrm3*, *Pde1a*, *Atp2b4*, *Glra1*, *Idi1*, *Fgf*, *Grin2b* and *Cda*. Total RNA was obtained from mice exposed to hypoxia conditions that were the same as those used in the microarray assay [44, 45]. Real-time RT-PCR was conducted in 20 μl reaction mixtures, including 10 μl Power SYBR Green PCR Master Mix (Applied Biosystems), 1 μl primers, 1 μl template cDNA, and 8 μl nuclease-free water. The cycling program consisted of a single cycle of 5 min at 95°C, followed by 40 cycles of 15 s at 95°C, and 1 min at 60°C (see Table 3). The optimized 2^−ΔΔCt^ method was used to calculate the relative gene expression. *Cacna2d1* was used as the internal control gene to normalize the amount of RNA added to the PCR reactions [15].

**Table 3:** The primer sequences for RT-PCR

Gene symbol	Direction	Primer sequence (5’-3’)	Product length (bp)
Cacna2d1	Forward	AGTTTATCCCAAAGAGGCCG	149
	Reverse	TTCCAGATTCATACGCACCAG	
Grin2a	Forward	TGGTGATCGTGCTGAATAAGG	147
	Reverse	AGGTGACAATGCTGAGGTG	
Npy1r	Forward	GCCCACTCTGCTTTATATTCATATG	108
	Reverse	ATTCGCTTGGTCTCACTGG	
Mef2c	Forward	CCAGATCTCCGCGTTCTTATC	149
	Reverse	CCTCCCATTCCTTGTCCTG	
Epha4	Forward	TCGTTATGTGGGAAGTGATGTC	133
	Reverse	AACTGATGGAGGGCAATGG	
Rxfp1	Forward	TGTGCTGGATTCCCATCTTC	148
	Reverse	AAAGGTCTAGTGGTCAACGTG	
Chrm3	Forward	TCGGTAGAGCGGACTGG	148
	Reverse	TTCACTCAATCCACAGTCCAC	
Pdela	Forward	TGCTATTCTGTACAACGACCG	148
	Reverse	GACCATTTCAATCACTAAGTTCCG	
Atp2b4	Forward	GGATTGGAGAACTTTTGTGGG	145
	Reverse	ATCTCGGCAAGGTCAATCTC	
Glral	Forward	GGAAGAGAAAGACCTGAGATACTG	144
	Reverse	GACAGGATGACGATGAGCAG	
Idil	Forward	ATTGGTGTGAAGCGAGCA	133
	Reverse	CACCCCAGATACCATCAGATTG	
Fgf1	Forward	TGGGACAAGGGACAGGAG	150
	Reverse	TCCTCATTTGGTGTCTGCG	
Grin2b	Forward	AAGGAGAGGAAGTGGGAGAG	128
	Reverse	AAGGTAACGATGCTCAGATGG	
Cda	Forward	CTGCCGACAAGTCATGAGAG	122
	Reverse	GGTCTTCAGGTCCAAACGAG	
β-actin	Forward	ACCTTCTACAATGAGCTGCG	146
	Reverse	CTGGATGGCTACGTACATGG	

## SUPPLEMENTARY MATERIALS TABLES






