# Supplementary material for: Identification and analysis of hub genes and networks related to hypoxia preconditioning in mice (No 035215)
Source: Oncotarget. 2017 Dec 21;9(15):11889–904. doi: 10.18632/oncotarget.23555 (PMC5844716; doi:10.18632/oncotarget.23555)
Supplement: Supplementary file 1 [file oncotarget-09-11889-s001.pdf]

Identification and analysis of hub genes and networks related to hypoxia preconditioning in mice

SUPPLEMENTARY MATERIALS

Supplementary Table 1: The significant functions of the 113 up-regulated differentially expressed genes. See_Supplementary_Table 1

Supplementary Table 2: The significant functions of the 138 down-regulated differentially expressed genes. See_Supplementary_Table 2

Supplementary Table 3: Nineteen of the up-regulated significant signaling pathways of the differentially expressed genes involved in HPC mice

Path id	Path name	*p*-value	FDR value	Enrichment
4080	Neuroactive ligand-receptor interaction	1.55E-12	6.49E-11	9.7600422
4020	Calcium signaling pathway	3.26E-06	6.85E-05	7.2030812
1100	Metabolic pathways	4.89E-05	6.84E-04	2.675919
4010	MAPK signaling pathway	0.0002841	2.39E-03	4.7400922
562	Inositol phosphate metabolism	0.0010964	6.58E-03	10.686753
4810	Regulation of actin cytoskeleton	0.0020076	9.65E-03	4.5312775
565	Ether lipid metabolism	0.0026809	1.12E-02	13.775893
5014	Amyotrophic lateral sclerosis (ALS)	0.0027207	1.13E-02	8.3967347
564	Glycerophospholipid metabolism	0.0035119	1.27E-02	7.8369524
4070	Phosphatidylinositol signaling system	0.0042499	1.37E-02	7.4401447
600	Sphingolipid metabolism	0.0063119	1.74E-02	10.251827
4530	Tight junction	0.0063321	1.75E-02	5.1021825
4144	Endocytosis	0.007971	1.97E-02	4.0442988
770	Pantothenate and CoA biosynthesis	0.0091502	2.13E-02	19.592381
4340	Hedgehog signaling pathway	0.0126362	2.79E-02	8.0150649
4740	Olfactory transduction	0.0180672	3.70E-02	0.1494841
340	Histidine metabolism	0.0249976	4.68E-02	11.755429
760	Nicotinate and nicotinamide metabolism	0.0249976	4.68E-02	11.755429
563	Glycosylphosphatidylinositol(GPI)-anchor biosynthesis	0.02696	4.92E-02	11.303297

Supplementary Table 4: Twenty-six of the down-regulated significant signaling pathways of the differentially expressed genes involved in HPC mice

Path id	Path name	*p*-value	FDR	Enrichment
4020	Calcium signaling pathway	1.27E-22	4.37E-21	14.181066
4080	Neuroactive ligand-receptor interaction	2.42E-19	4.16E-18	10.675046
4720	Long-term potentiation	5.73E-10	4.93E-09	14.550669
4360	Axon guidance	1.77E-08	1.22E-07	9.25
4310	Wnt signaling pathway	9.86E-08	5.65E-07	7.9367284
4070	Phosphatidylinositol signaling system	1.60E-06	7.87E-06	10.850211
4540	Gap junction	6.54E-06	2.81E-05	9.022807
561	Glycerolipid metabolism	1.82E-05	6.62E-05	12.362981
4810	Regulation of actin cytoskeleton	2.25E-05	7.73E-05	5.1920888
4062	Chemokine signaling pathway	4.87E-05	0.000148	5.2781199
4260	Cardiac muscle contraction	6.47E-05	0.000183	7.8949561
4270	Vascular smooth muscle contraction	8.17E-05	0.000216	6.3967662
4912	GnRH signaling pathway	0.0001088	0.000267	7.2817557
4520	Adherens junction	0.0002011	0.000461	8.1376582
230	Purine metabolism	0.0005019	0.001079	4.9262452
4510	Focal adhesion	0.0015847	0.002849	4.1409018
4730	Long-term depression	0.0016776	0.002974	6.8683226
4370	VEGF signaling pathway	0.0018815	0.003237	6.6966146
601	Glycosphingolipid biosynthesis – lacto and neolacto series	0.0028290	0.004636	13.393229
4740	Olfactory transduction	0.0100608	0.013589	0.2179976
4115	p53 signaling pathway	0.0113423	0.014857	5.6392544
4670	Leukocyte transendothelial migration	0.0118037	0.015296	4.3912227
4710	Circadian rhythm – mammal	0.0146925	0.017838	15.306548
4650	Natural killer cell mediated cytotoxicity	0.0158370	0.018759	4.0895356
4662	B cell receptor signaling pathway	0.0167677	0.019475	5.0421569
604	Glycosphingolipid biosynthesis – ganglio series	0.0191375	0.021243	13.393229
4012	ErbB signaling pathway	0.0228182	0.023936	4.6084229
562	Inositol phosphate metabolism	0.0296356	0.029142	5.8443182
