# Supplementary material for: Identification and analysis of hub genes and networks related to hypoxia preconditioning in mice (No 035215)
Source: Oncotarget. 2017 Dec 21;9(15):11889–904. doi: 10.18632/oncotarget.23555 (PMC5844716; doi:10.18632/oncotarget.23555)
Supplement: Supplementary file 2 [file oncotarget-09-11889-s002.doc]

**Supplementary Table 1:** **The significant functions of the 113 up-regulated differentially expressed genes**

| Go id | Go name | *p*-value | FDR | | Enrichment | |
| --- | --- | --- | --- | --- | --- | --- |
| GO:0006811 | ion transport | 4.2E-07 | | 4.0E-05 | | 3.35302 |
| GO:0006836 | neurotransmitter transport | 7.8E-06 | | 0.00028 | | 10.57919 |
| GO:0045110 | intermediate filament bundle assembly | 3.3E-05 | | 0.00091 | | 46.47289 |
| GO:0009636 | response to toxin | 0.00010 | | 0.00224 | | 7.22912 |
| GO:0045104 | intermediate filament cytoskeleton organization | 0.00016 | | 0.00317 | | 30.98193 |
| GO:0060216 | definitive hemopoiesis | 0.00044 | | 0.00577 | | 23.23645 |
| GO:0021937 | cerebellar Purkinje cell-granule cell precursor cell signaling involved in regulation of granule cell precursor cell proliferation | 0.00052 | | 0.00622 | | 61.96386 |
| GO:0007268 | synaptic transmission | 0.00055 | | 0.00634 | | 5.56086 |
| GO:0042493 | response to drug | 0.00057 | | 0.00647 | | 3.17138 |
| GO:0060052 | neurofilament cytoskeleton organization | 0.00066 | | 0.00682 | | 20.65462 |
| GO:0007165 | signal transduction | 0.00076 | | 0.00719 | | 2.44764 |
| GO:0014070 | response to organic cyclic substance | 0.00081 | | 0.00739 | | 4.54781 |
| GO:0010811 | positive regulation of cell-substrate adhesion | 0.00111 | | 0.00905 | | 10.32731 |
| GO:0000226 | microtubule cytoskeleton organization | 0.00112 | | 0.00913 | | 7.37665 |
| GO:0031133 | regulation of axon diameter | 0.00154 | | 0.01093 | | 41.30924 |
| GO:0045103 | intermediate filament-based process | 0.00154 | | 0.01093 | | 41.30924 |
| GO:0009612 | response to mechanical stimulus | 0.00155 | | 0.01096 | | 6.88487 |
| GO:0051289 | protein homotetramerization | 0.00203 | | 0.01273 | | 8.85198 |
| GO:0032526 | response to retinoic acid | 0.00209 | | 0.01293 | | 6.45457 |
| GO:0046887 | positive regulation of hormone secretion | 0.00212 | | 0.01304 | | 14.29935 |
| GO:0007568 | aging | 0.00211 | | 0.01317 | | 4.42599 |
| GO:0007267 | cell-cell signaling | 0.00252 | | 0.01421 | | 6.19639 |
| GO:0021545 | cranial nerve development | 0.00306 | | 0.01590 | | 30.98193 |
| GO:0007218 | neuropeptide signaling pathway | 0.00356 | | 0.01740 | | 4.70612 |
| GO:0042127 | regulation of cell proliferation | 0.00361 | | 0.01753 | | 4.05371 |
| GO:0042552 | myelination | 0.00380 | | 0.01806 | | 7.51077 |
| GO:0055085 | transmembrane transport | 0.00456 | | 0.0199 | | 2.20374 |
| GO:0045941 | positive regulation of transcription | 0.00471 | | 0.02022 | | 3.46651 |
| GO:0048016 | inositol phosphate-mediated signaling | 0.00504 | | 0.02091 | | 24.78554 |
| GO:0006629 | lipid metabolic process | 0.00512 | | 0.02109 | | 3.41870 |
| GO:0006821 | chloride transport | 0.00527 | | 0.02136 | | 6.88487 |
| GO:0042391 | regulation of membrane potential | 0.00527 | | 0.02136 | | 6.88487 |
| GO:0006813 | potassium ion transport | 0.00741 | | 0.02558 | | 3.21890 |
| GO:0006729 | tetrahydrobiopterin biosynthetic process | 0.00748 | | 0.02568 | | 20.65462 |
| GO:0007204 | elevation of cytosolic calcium ion concentration | 0.00917 | | 0.02821 | | 4.62417 |
| GO:0007155 | cell adhesion | 0.00995 | | 0.02919 | | 2.15152 |
| GO:0007160 | cell-matrix adhesion | 0.01010 | | 0.02937 | | 5.76408 |
| GO:0051592 | response to calcium ion | 0.01010 | | 0.02937 | | 5.76408 |
| GO:0014065 | phosphoinositide 3-kinase cascade | 0.01036 | | 0.02967 | | 17.70396 |
| GO:0032270 | positive regulation of cellular protein metabolic process | 0.01036 | | 0.02967 | | 17.70396 |
| GO:0045598 | regulation of fat cell differentiation | 0.01036 | | 0.02967 | | 17.70396 |
| GO:0060484 | lung-associated mesenchyme development | 0.01036 | | 0.02967 | | 17.70396 |
| GO:0048008 | platelet-derived growth factor receptor signaling pathway | 0.01167 | | 0.03108 | | 8.08224 |
| GO:0000160 | two-component signal transduction system (phosphorelay) | 0.01366 | | 0.03287 | | 15.49096 |
| GO:0019229 | regulation of vasoconstriction | 0.01366 | | 0.03287 | | 15.49096 |
| GO:0021702 | cerebellar Purkinje cell differentiation | 0.01366 | | 0.03287 | | 15.49096 |
| GO:0030041 | actin filament polymerization | 0.01366 | | 0.03287 | | 15.49096 |
| GO:0007243 | intracellular protein kinase cascade | 0.01396 | | 0.03312 | | 4.18675 |
| GO:0006915 | apoptosis | 0.01462 | | 0.03362 | | 2.19490 |
| GO:0032870 | cellular response to hormone stimulus | 0.01479 | | 0.03375 | | 7.43566 |
| GO:0045768 | positive regulation of anti-apoptosis | 0.01479 | | 0.03375 | | 7.43566 |
| GO:0007417 | central nervous system development | 0.01725 | | 0.03537 | | 4.95711 |
| GO:0015813 | L-glutamate transport | 0.01738 | | 0.03544 | | 13.76975 |
| GO:0051789 | response to protein stimulus | 0.01849 | | 0.03606 | | 4.85991 |
| GO:0048146 | positive regulation of fibroblast proliferation | 0.02033 | | 0.03698 | | 6.63898 |
| GO:0015074 | DNA integration | 0.02149 | | 0.03750 | | 12.39277 |
| GO:0051781 | positive regulation of cell division | 0.02460 | | 0.03870 | | 6.19639 |
| GO:0000122 | negative regulation of transcription from RNA polymerase II promoter | 0.02541 | | 0.03897 | | 2.43526 |
| GO:0001666 | response to hypoxia | 0.02580 | | 0.03910 | | 2.81654 |
| GO:0042176 | regulation of protein catabolic process | 0.02599 | | 0.03916 | | 11.26616 |
| GO:0030890 | positive regulation of B cell proliferation | 0.02692 | | 0.03945 | | 5.99650 |
| GO:0007399 | nervous system development | 0.02817 | | 0.03981 | | 2.39345 |
| GO:0001525 | angiogenesis | 0.02906 | | 0.04006 | | 3.04740 |
| GO:0042572 | retinol metabolic process | 0.03085 | | 0.04051 | | 10.32731 |
| GO:0045765 | regulation of angiogenesis | 0.03085 | | 0.04051 | | 10.32731 |
| GO:0045773 | positive regulation of axon extension | 0.03085 | | 0.04051 | | 10.32731 |
| GO:0045987 | positive regulation of smooth muscle contraction | 0.03085 | | 0.04051 | | 10.32731 |
| GO:0001999 | renal response to blood flow involved in circulatory 2ynthe-angiotensin regulation of systemic arterial blood pressure | 0.03228 | | 0.04085 | | 61.96386 |
| GO:0002041 | intussusceptive angiogenesis | 0.03228 | | 0.04085 | | 61.96386 |
| GO:0006288 | base-excision repair, DNA ligation | 0.03228 | | 0.04085 | | 61.96386 |
| GO:0006507 | GPI anchor release | 0.03228 | | 0.04085 | | 61.96386 |
| GO:0006581 | acetylcholine catabolic process | 0.03228 | | 0.04085 | | 61.96386 |
| GO:0006587 | serotonin biosynthetic process from tryptophan | 0.03228 | | 0.04085 | | 61.96386 |
| GO:0006760 | folic acid and derivative metabolic process | 0.03228 | | 0.04085 | | 61.96386 |
| GO:0006837 | serotonin transport | 0.03228 | | 0.04085 | | 61.96386 |
| GO:0006931 | substrate-dependent cell migration, cell attachment to substrate | 0.03228 | | 0.04085 | | 61.96386 |
| GO:0008065 | establishment of blood-nerve barrier | 0.03228 | | 0.04085 | | 61.96386 |
| GO:0008292 | acetylcholine biosynthetic process | 0.03228 | | 0.04085 | | 61.96386 |
| GO:0009134 | nucleoside diphosphate catabolic process | 0.03228 | | 0.04085 | | 61.96386 |
| GO:0009214 | cyclic nucleotide catabolic process | 0.03228 | | 0.04085 | | 61.96386 |
| GO:0009258 | 10-formyltetrahydrofolate catabolic process | 0.03228 | | 0.04085 | | 61.96386 |
| GO:0009972 | cytidine deamination | 0.03228 | | 0.04085 | | 61.96386 |
| GO:0014873 | response to muscle activity involved in regulation of muscle adaptation | 0.03228 | | 0.04085 | | 61.96386 |
| GO:0015868 | purine ribonucleotide transport | 0.03228 | | 0.04085 | | 61.96386 |
| GO:0016117 | carotenoid biosynthetic process | 0.03228 | | 0.04085 | | 61.96386 |
| GO:0030185 | nitric oxide transport | 0.03228 | | 0.04085 | | 61.96386 |
| GO:0031109 | microtubule polymerization or depolymerization | 0.03228 | | 0.04085 | | 61.96386 |
| GO:0031344 | regulation of cell projection organization | 0.03228 | | 0.04085 | | 61.96386 |
| GO:0031583 | activation of phospholipase D by G-protein coupled receptor protein signaling pathway | 0.03228 | | 0.04085 | | 61.96386 |
| GO:0032075 | positive regulation of nuclease activity | 0.03228 | | 0.04085 | | 61.96386 |
| GO:0033157 | regulation of intracellular protein transport | 0.03228 | | 0.04085 | | 61.96386 |
| GO:0033693 | neurofilament bundle assembly | 0.03228 | | 0.04085 | | 61.96386 |
| GO:0034120 | positive regulation of erythrocyte aggregation | 0.03228 | | 0.04085 | | 61.96386 |
| GO:0034126 | positive regulation of MyD88-dependent toll-like receptor signaling pathway | 0.03228 | | 0.04085 | | 61.96386 |
| GO:0035499 | carnosine biosynthetic process | 0.03228 | | 0.04085 | | 61.96386 |
| GO:0042219 | cellular amino acid derivative catabolic process | 0.03228 | | 0.04085 | | 61.96386 |
| GO:0042313 | protein kinase C deactivation | 0.03228 | | 0.04085 | | 61.96386 |
| GO:0043006 | activation of phospholipase A2 activity by calcium-mediated signaling | 0.03228 | | 0.04085 | | 61.96386 |
| GO:0045212 | neurotransmitter receptor biosynthetic process | 0.03228 | | 0.04085 | | 61.96386 |
| GO:0046087 | cytidine metabolic process | 0.03228 | | 0.04085 | | 61.96386 |
| GO:0046836 | glycolipid transport | 0.03228 | | 0.04085 | | 61.96386 |
| GO:0051610 | serotonin uptake | 0.03228 | | 0.04085 | | 61.96386 |
| GO:0051771 | negative regulation of nitric-oxide synthase biosynthetic process | 0.03228 | | 0.04085 | | 61.96386 |
| GO:0060123 | regulation of growth hormone secretion | 0.03228 | | 0.04085 | | 61.96386 |
| GO:0060213 | positive regulation of nuclear-transcribed mRNA poly(A) tail shortening | 0.03228 | | 0.04085 | | 61.96386 |
| GO:0071206 | establishment of protein localization to juxtaparanode region of axon | 0.03228 | | 0.04085 | | 61.96386 |
| GO:0071345 | cellular response to cytokine stimulus | 0.03228 | | 0.04085 | | 61.96386 |
| GO:0090131 | mesenchyme migration | 0.03228 | | 0.04085 | | 61.96386 |
| GO:0001508 | regulation of action potential | 0.03607 | | 0.04337 | | 9.53290 |
| GO:0010243 | response to organic nitrogen | 0.03738 | | 0.04419 | | 5.31119 |
| GO:0043065 | positive regulation of apoptosis | 0.04399 | | 0.04791 | | 2.77450 |
| GO:0042632 | cholesterol homeostasis | 0.04651 | | 0.04918 | | 4.89188 |
| GO:0007274 | neuromuscular synaptic transmission | 0.04754 | | 0.04968 | | 8.26185 |
