# Supplementary material for: Identification and analysis of hub genes and networks related to hypoxia preconditioning in mice (No 035215)
Source: Oncotarget. 2017 Dec 21;9(15):11889–904. doi: 10.18632/oncotarget.23555 (PMC5844716; doi:10.18632/oncotarget.23555)
Supplement: Supplementary file 3 [file oncotarget-09-11889-s003.doc]

**Supplementary Table 2: The significant functions of the 138 down-regulated differentially expressed genes**

| Go id | Go name | *p*-value | FDR | Enrichment |
| --- | --- | --- | --- | --- |
| GO:0006813 | potassium ion transport | 1.04E-18 | 2.3E-16 | 10.46143 |
| GO:0006811 | ion transport | 4.07E-18 | 4.4E-16 | 5.49896 |
| GO:0006816 | calcium ion transport | 4.87E-12 | 3.5E-10 | 9.66409 |
| GO:0007165 | signal transduction | 1.11E-11 | 6.0E-10 | 4.25116 |
| GO:0007399 | nervous system development | 3.73E-09 | 1.6E-07 | 5.31879 |
| GO:0000160 | two-component signal transduction system (phosphorelay) | 1.17E-07 | 2.8E-06 | 38.72741 |
| GO:0007264 | small GTPase mediated signal transduction | 3.21E-06 | 7.0E-05 | 4.81941 |
| GO:0007420 | brain development | 1.09E-05 | 0.00019 | 5.54148 |
| GO:0023034 | intracellular signaling pathway | 1.61E-05 | 0.00025 | 4.85991 |
| GO:0006355 | regulation of transcription, DNA-dependent | 2.64E-05 | 0.00035 | 2.22434 |
| GO:0048169 | regulation of long-term neuronal synaptic plasticity | 2.75E-05 | 0.00035 | 15.49096 |
| GO:0001764 | neuron migration | 2.81E-05 | 0.00036 | 7.28987 |
| GO:0060079 | regulation of excitatory postsynaptic membrane potential | 3.56E-05 | 0.00041 | 14.75330 |
| GO:0045471 | response to ethanol | 5.81E-05 | 0.00060 | 6.60948 |
| GO:0008217 | regulation of blood pressure | 7.21E-05 | 0.00069 | 9.53290 |
| GO:0007268 | synaptic transmission | 7.74E-05 | 0.00073 | 6.35527 |
| GO:0051056 | regulation of small GTPase mediated signal transduction | 8.20E-05 | 0.00076 | 7.47840 |
| GO:0007411 | axon guidance | 0.00011 | 0.00097 | 6.04525 |
| GO:0048715 | negative regulation of oligodendrocyte differentiation | 0.00016 | 0.00133 | 30.98193 |
| GO:0007205 | activation of protein kinase C activity by G-protein coupled receptor protein signaling pathway | 0.00022 | 0.00171 | 10.32731 |
| GO:0030182 | neuron differentiation | 0.00023 | 0.00177 | 6.37863 |
| GO:0051968 | positive regulation of synaptic transmission, glutamatergic | 0.00028 | 0.00205 | 26.55594 |
| GO:0021952 | central nervous system projection neuron axonogenesis | 0.00044 | 0.00281 | 23.23645 |
| GO:0007158 | neuron cell-cell adhesion | 0.00052 | 0.00311 | 61.96386 |
| GO:0021882 | regulation of transcription from RNA polymerase II promoter involved in forebrain neuron fate commitment | 0.00052 | 0.00311 | 61.96386 |
| GO:0021893 | cerebral cortex GABAergic interneuron fate commitment | 0.00052 | 0.00311 | 61.96386 |
| GO:0001975 | response to amphetamine | 0.00053 | 0.00315 | 12.39277 |
| GO:0048167 | regulation of synaptic plasticity | 0.00078 | 0.00432 | 11.26616 |
| GO:0050679 | positive regulation of epithelial cell proliferation | 0.00089 | 0.00480 | 7.74548 |
| GO:0035235 | ionotropic glutamate receptor signaling pathway | 0.00092 | 0.00492 | 18.58916 |
| GO:0009968 | negative regulation of signal transduction | 0.00126 | 0.00619 | 7.20510 |
| GO:0007204 | elevation of cytosolic calcium ion concentration | 0.00150 | 0.00702 | 5.54900 |
| GO:0007409 | axonogenesis | 0.00150 | 0.00702 | 5.54900 |
| GO:0060078 | regulation of postsynaptic membrane potential | 0.00154 | 0.00715 | 41.30924 |
| GO:0051924 | regulation of calcium ion transport | 0.00165 | 0.00750 | 15.49096 |
| GO:0001508 | regulation of action potential | 0.00212 | 0.00903 | 14.29935 |
| GO:0016199 | axon midline choice point recognition | 0.00306 | 0.01159 | 30.98193 |
| GO:0021892 | cerebral cortex GABAergic interneuron differentiation | 0.00306 | 0.01159 | 30.98193 |
| GO:0048755 | branching morphogenesis of a nerve | 0.00306 | 0.01159 | 30.98193 |
| GO:0060087 | relaxation of vascular smooth muscle | 0.00306 | 0.01159 | 30.98193 |
| GO:0019228 | regulation of action potential in neuron | 0.00330 | 0.01216 | 12.39277 |
| GO:0050804 | regulation of synaptic transmission | 0.00330 | 0.01216 | 12.39277 |
| GO:0007218 | neuropeptide signaling pathway | 0.00356 | 0.01275 | 4.70612 |
| GO:0007156 | ynthesize cell adhesion | 0.00394 | 0.01355 | 3.56627 |
| GO:0006937 | regulation of muscle contraction | 0.00401 | 0.01369 | 11.61822 |
| GO:0046777 | protein amino acid autophosphorylation | 0.00405 | 0.01375 | 4.58992 |
| GO:0048168 | regulation of neuronal synaptic plasticity | 0.00481 | 0.01559 | 10.93480 |
| GO:0007213 | muscarinic acetylcholine receptor signaling pathway | 0.00504 | 0.01610 | 24.78554 |
| GO:0014061 | regulation of norepinephrine secretion | 0.00504 | 0.01610 | 24.78554 |
| GO:0042220 | response to cocaine | 0.00571 | 0.01753 | 10.32731 |
| GO:0045776 | negative regulation of blood pressure | 0.00571 | 0.01753 | 10.32731 |
| GO:0043267 | negative regulation of potassium ion transport | 0.00748 | 0.02083 | 20.65462 |
| GO:0045921 | positive regulation of exocytosis | 0.00748 | 0.02083 | 20.65462 |
| GO:0048675 | axon extension | 0.00748 | 0.02083 | 20.65462 |
| GO:0051001 | negative regulation of nitric-oxide synthase activity | 0.00748 | 0.02083 | 20.65462 |
| GO:0001934 | positive regulation of protein amino acid phosphorylation | 0.00777 | 0.02132 | 6.19639 |
| GO:0000122 | negative regulation of transcription from RNA polymerase II promoter | 0.00872 | 0.02280 | 2.70585 |
| GO:0045907 | positive regulation of vasoconstriction | 0.00897 | 0.02317 | 8.85198 |
| GO:0035023 | regulation of Rho protein signal transduction | 0.00917 | 0.02346 | 4.62417 |
| GO:0007413 | axonal fasciculation | 0.01036 | 0.02505 | 17.70396 |
| GO:0014049 | positive regulation of glutamate secretion | 0.01036 | 0.02505 | 17.70396 |
| GO:0032230 | positive regulation of synaptic transmission, GABAergic | 0.01036 | 0.02505 | 17.70396 |
| GO:0050966 | detection of mechanical stimulus involved in sensory perception of pain | 0.01036 | 0.02505 | 17.70396 |
| GO:0019216 | regulation of lipid metabolic process | 0.01366 | 0.02871 | 15.49096 |
| GO:0019432 | triglyceride biosynthetic process | 0.01366 | 0.02871 | 15.49096 |
| GO:0031175 | neuron projection development | 0.01607 | 0.03083 | 5.05827 |
| GO:0007154 | cell communication | 0.01736 | 0.03182 | 3.97204 |
| GO:0008284 | positive regulation of cell proliferation | 0.01806 | 0.03232 | 2.42996 |
| GO:0006486 | protein amino acid glycosylation | 0.01978 | 0.03344 | 4.76645 |
| GO:0007169 | transmembrane receptor protein tyrosine kinase signaling pathway | 0.02112 | 0.03425 | 4.67652 |
| GO:0021879 | forebrain neuron differentiation | 0.02149 | 0.03445 | 12.39277 |
| GO:0021953 | central nervous system neuron differentiation | 0.02149 | 0.03446 | 12.39277 |
| GO:0045600 | positive regulation of fat cell differentiation | 0.02149 | 0.03446 | 12.39277 |
| GO:0007155 | cell adhesion | 0.02293 | 0.03524 | 2.00809 |
| GO:0006470 | protein amino acid dephosphorylation | 0.02311 | 0.03532 | 3.20503 |
| GO:0006874 | cellular calcium ion homeostasis | 0.02551 | 0.03647 | 4.42599 |
| GO:0042417 | dopamine metabolic process | 0.02598 | 0.03668 | 11.26616 |
| GO:0048041 | focal adhesion assembly | 0.02598 | 0.03668 | 11.26616 |
| GO:0001649 | osteoblast differentiation | 0.02935 | 0.03802 | 5.80911 |
| GO:0007189 | activation of adenylate cyclase activity by G-protein signaling pathway | 0.02935 | 0.03802 | 5.80911 |
| GO:0045944 | positive regulation of transcription from RNA polymerase II promoter | 0.03072 | 0.03850 | 2.05974 |
| GO:0001964 | startle response | 0.03085 | 0.03854 | 10.32731 |
| GO:0007271 | synaptic transmission, cholinergic | 0.03085 | 0.03854 | 10.32731 |
| GO:0031000 | response to caffeine | 0.03085 | 0.03854 | 10.32731 |
| GO:0045987 | positive regulation of smooth muscle contraction | 0.03085 | 0.03854 | 10.32731 |
| GO:0051145 | smooth muscle cell differentiation | 0.03085 | 0.03854 | 10.32731 |
| GO:0051605 | protein maturation by peptide bond cleavage | 0.03085 | 0.03854 | 10.32731 |
| GO:0001821 | histamine secretion | 0.03228 | 0.03901 | 61.96386 |
| GO:0002209 | behavioral defense response | 0.03228 | 0.03901 | 61.96386 |
| GO:0002674 | negative regulation of acute inflammatory response | 0.03228 | 0.03901 | 61.96386 |
| GO:0002866 | positive regulation of acute inflammatory response to antigenic stimulus | 0.03228 | 0.03901 | 61.96386 |
| GO:0007197 | inhibition of adenylate cyclase activity by muscarinic acetylcholine receptor signaling pathway | 0.03228 | 0.03901 | 61.96386 |
| GO:0007207 | activation of phospholipase C activity by muscarinic acetylcholine receptor signaling pathway | 0.03228 | 0.03901 | 61.96386 |
| GO:0009069 | serine family amino acid metabolic process | 0.03228 | 0.03901 | 61.96386 |
| GO:0014057 | positive regulation of acetylcholine secretion | 0.03228 | 0.03901 | 61.96386 |
| GO:0016081 | synaptic vesicle docking involved in exocytosis | 0.03228 | 0.03901 | 61.96386 |
| GO:0021572 | rhombomere 6 development | 0.03228 | 0.03901 | 61.96386 |
| GO:0021885 | forebrain cell migration | 0.03228 | 0.03901 | 61.96386 |
| GO:0030322 | stabilization of membrane potential | 0.03228 | 0.03901 | 61.96386 |
| GO:0031915 | positive regulation of synaptic plasticity | 0.03228 | 0.03901 | 61.96386 |
| GO:0032011 | ARF protein signal transduction | 0.03228 | 0.03901 | 61.96386 |
| GO:0032317 | regulation of Rap GTPase activity | 0.03228 | 0.03901 | 61.96386 |
| GO:0032344 | regulation of aldosterone metabolic process | 0.03228 | 0.03901 | 61.96386 |
| GO:0032461 | positive regulation of protein oligomerization | 0.03228 | 0.03901 | 61.96386 |
| GO:0032861 | activation of Rap GTPase activity | 0.03228 | 0.03901 | 61.96386 |
| GO:0034231 | islet amyloid polypeptide processing | 0.03228 | 0.03901 | 61.96386 |
| GO:0034465 | response to carbon monoxide | 0.03228 | 0.03901 | 61.96386 |
| GO:0034633 | retinol transport | 0.03228 | 0.03901 | 61.96386 |
| GO:0035408 | histone H3-T6 phosphorylation | 0.03228 | 0.03901 | 61.96386 |
| GO:0042137 | sequestering of neurotransmitter | 0.03228 | 0.03901 | 61.96386 |
| GO:0042339 | 4ynthes sulfate metabolic process | 0.03228 | 0.03901 | 61.96386 |
| GO:0042376 | phylloquinone catabolic process | 0.03228 | 0.03901 | 61.96386 |
| GO:0042976 | activation of Janus kinase activity | 0.03228 | 0.03901 | 61.96386 |
| GO:0043624 | cellular protein complex disassembly | 0.03228 | 0.03901 | 61.96386 |
| GO:0045428 | regulation of nitric oxide biosynthetic process | 0.03228 | 0.03901 | 61.96386 |
| GO:0045713 | low-density lipoprotein receptor biosynthetic process | 0.03228 | 0.03901 | 61.96386 |
| GO:0046636 | negative regulation of alpha-beta T cell activation | 0.03228 | 0.03901 | 61.96386 |
| GO:0051041 | positive regulation of calcium-independent cell-cell adhesion | 0.03228 | 0.03901 | 61.96386 |
| GO:0051533 | positive regulation of NFAT protein import into nucleus | 0.03228 | 0.03901 | 61.96386 |
| GO:0051835 | positive regulation of synapse structural plasticity | 0.03228 | 0.03901 | 61.96386 |
| GO:0052565 | response to defense-related host nitric oxide production | 0.03228 | 0.03901 | 61.96386 |
| GO:0060004 | reflex | 0.03228 | 0.03901 | 61.96386 |
| GO:0070179 | D-serine biosynthetic process | 0.03228 | 0.03901 | 61.96386 |
| GO:0070327 | thyroid hormone transport | 0.03228 | 0.03901 | 61.96386 |
| GO:0090073 | positive regulation of protein homodimerization activity | 0.03228 | 0.03901 | 61.96386 |
| GO:0042311 | vasodilation | 0.03607 | 0.04184 | 9.53290 |
| GO:0045664 | regulation of neuron differentiation | 0.03607 | 0.03901 | 9.53290 |
| GO:0030178 | negative regulation of Wnt receptor signaling pathway | 0.03738 | 0.04276 | 5.31119 |
| GO:0000082 | G1/S transition of mitotic cell cycle | 0.04030 | 0.04473 | 5.16365 |
| GO:0042391 | regulation of membrane potential | 0.04030 | 0.04473 | 5.16365 |
| GO:0007188 | G-protein signaling, coupled to cAMP nucleotide second messenger | 0.04164 | 0.04559 | 8.85198 |
| GO:0007193 | inhibition of adenylate cyclase activity by G-protein signaling pathway | 0.04164 | 0.04559 | 8.85198 |
| GO:0018107 | peptidyl-threonine phosphorylation | 0.04164 | 0.04559 | 8.85198 |
| GO:0050729 | positive regulation of inflammatory response | 0.04164 | 0.04559 | 8.85198 |
| GO:0043065 | positive regulation of apoptosis | 0.04399 | 0.04705 | 2.77450 |
| GO:0030900 | forebrain development | 0.04417 | 0.04716 | 3.75539 |
| GO:0007274 | neuromuscular synaptic transmission | 0.04754 | 0.04913 | 8.26185 |
| GO:0031290 | retinal ganglion cell axon guidance | 0.04754 | 0.04913 | 8.26185 |
